# Mediators of the effect of the JUMP-in intervention on physical activity and sedentary behavior in Dutch primary schoolchildren from disadvantaged neighborhoods

**DOI:** 10.1186/1479-5868-9-131

**Published:** 2012-11-06

**Authors:** Maartje M van Stralen, Judith de Meij, Saskia J te Velde, Marcel F van der Wal, Willem van Mechelen, Dirk L Knol, Mai JM Chinapaw

**Affiliations:** 1 EMGO Institute for Health and Care Research and the Department of Public and Occupational Health, VU University Medical Center, Amsterdam, The Netherlands; 2 Department of Epidemiology, Documentation and Health Promotion, Municipal Health Service Amsterdam, Amsterdam, The Netherlands; 3 EMGO Institute for Health and Care Research and the Department of Epidemiology and Biostatistics, VU University Medical Center, Amsterdam, The Netherlands

**Keywords:** Mediating, Working mechanisms, Youth, Intervention, Physical activity, Sport, Sedentary, Sitting, Television

## Abstract

**Background:**

Important health benefits can be achieved when physical activity in children from low socio-economic status is promoted and sedentariness is limited. By specifying the mediating mechanisms of existing interventions one can improve future physical activity interventions. This study explored potential mediators of the long-term effect of the school-based multicomponent JUMP-in intervention on sport participation, outdoor play and screen time in Dutch primary schoolchildren from disadvantaged neighborhoods.

**Methods:**

In total, 600 primary schoolchildren (aged 9.8 ± 0.7, 51% girls, 13% Dutch ethnicity, 35% overweight) from 9 intervention and 10 control schools were included in the analyses. JUMP-in was developed using Intervention Mapping, and targeted psychological and environmental determinants of physical activity. Outcome behaviors were self-reported sport participation, outdoor play, TV-viewing behavior and computer use. Potential mediators were self-reported psychological, social and physical environmental factors.

**Results:**

JUMP-in was effective in improving sport participation after 20 months, but not in improving outdoor play, or reducing TV-viewing or computer time. JUMP-in was not effective in changing hypothesized mediators so no significant mediated effects could be identified. However, changes in self-efficacy, social support and habit strength were positively associated with changes in sport participation, and changes in social support, self-efficacy, perceived planning skills, enjoyment and habit strength were positively associated with changes in outdoor play. Changes in enjoyment was positively associated with changes in TV-viewing while parental rules were negatively associated. Having a computer in the bedroom and enjoyment were positively associated with changes in computer use, while changes in parental rules were negatively associated.

**Conclusions:**

Besides a significant positive effect on sports participation, no significant intervention effect on outdoor play, screen time or any of the potential mediators was found. This suggest that other (unmeasured) factors operated as mediating mechanisms of the intervention, that we used unsuccessful intervention strategies, that the strategies were inappropriately implemented, or that children are unable to accurately recall past activities and cognitions. Additionally, the school setting might not be the sole channel to influence leisure time activities. Still, several personal and environmental constructs were found to be relevant in predicting change in sport participation, outdoor play and screen behavior and seem to be potential mediators. Future interventions are recommended including more effective strategies targeting these relevant constructs, addressing different constructs (e.g. pedagogic skills of parents), and focusing on different implementation settings.

**Trail registration:**

ISRCTN17489378

## Introduction

Regular physical activity (PA) and low levels of sedentary behavior (SB) have been associated with a decreased risk of physical and mental health problems [1-5]. Participation in physical and sedentary activities have a strong socio-economic and ethnic gradient, with children from a low socio-economic status or from an ethnic minority being less likely to participate in regular PA and more likely to be sedentary [6-13]. Important health benefits can be achieved when regular PA in children from low socio-economic status or ethnic minorities is promoted, initiation of activity of inactive children is encouraged and sedentary time is limited.

Schools have been identified as important arenas for PA promotion in young people. While school-based obesity prevention interventions were to some degree effective in changing PA, effect sizes were small [14,15]. To increase their effectiveness knowledge of effective mechanisms underlying PA behavior change is needed [16]. By conducting mediation analysis one can gain insight into which mechanisms are critical for influencing PA, e.g. insight into whether the intervention affected the potential mediator and whether this in turn affected the behavior [17]. This insight into what works and what does not work in interventions informs future intervention development and can improve their (cost)-effectiveness [18]. Even in the absence of a significant main effect on the behavior, these so-called mediation analysis should be conducted as it unfolds why the intervention was ineffective in changing behaviors, and how the intervention should be adapted to increase its effectiveness. Consequently, mediation analyses will not only increase the effectiveness of future interventions, but they will also help to reduce their costs [18] and will add to our understanding of behavior change.

A systematic review found strong evidence for the mediating effect of self-efficacy on the effect of interventions on PA, while moderate evidence was found for the mediating effect of intention. The evidence with regard to mediators of intervention effects on sedentary behavior is limited and inconclusive [19]. To date most overweight prevention intervention studies analyzed the mediating effect of personal determinants (e.g. self-efficacy, intention), whereas studies examining mediating effects of changes in the home and school environmental are largely lacking. In addition, all except one SB study [20] were conducted among secondary schoolchildren, limiting the generelizability of the findings to other age groups, such as primary schoolchildren. Therefore, more studies assessing mediators among primary schoolchildren are needed, especially on potential environmental mediators and potential mediators of sedentary behavior interventions.

**Figure 1:**
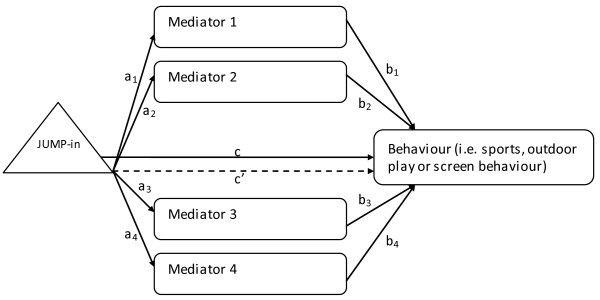
Conceptual mediation model.

The JUMP-in intervention is a theory, practice and evidence based primary school-based intervention aimed at improving PA in primary schoolchildren living in disadvantaged areas in Amsterdam, the Netherlands [21]. The intervention proved effective in changing the primary outcome organized sports participation [22]. The aims of the present study were 1) to examine the JUMP-in intervention effects on outdoor play and screen behavior; and 2) to conduct secondary data analysis to examine whether changes in personal (e.g. attitude, self-efficacy, intention, perceived planning skills) and environmental determinants (e.g. social modeling, social pressure, social norm, social support, perceived barriers) mediated the effect of the intervention on sport participation, outdoor play and screen behaviors (See Figure 1). The intervention was developed to target all of these underlying constructs, and it was hypothesized that these constructs would act as mediators in predicting changes in sport participation, outdoor play and screen behaviors.

## Methods

This study is registered at the Dutch Trial Register (Trial registration: ISRCTN17489378 ) and the protocol was approved by the Medical Ethics Committee of VU University Medical Center.

### Procedure and participants

This two-year controlled trial was carried out in nineteen primary schools situated in disadvantaged areas in Amsterdam, the Netherlands. A total of 708 boys and girls from grades 6 and 7 (aged 8–12) participated in the trial, and were interviewed about their sport participation and completed questionnaires on participation in outdoor play, screen behaviors and their potential mediators. Nine intervention schools were recruited in two city districts in Amsterdam, the Netherlands. Ten comparable control schools were recruited from geographically separated city districts to limit the possibility of contamination between intervention and control schools. Random assignment of schools to a control or intervention group was not possible because of prolonged preparations needed for a successful adoption and implementation of JUMP-in: a school and environmental scan had to be carried out and commitment had to be built among school staff and local partners in sports, care and prevention. Further, networks had to be created among participating organizations, and organizational practices had to be prepared for the implementation of the program and related protocols. The control schools were asked to continue their usual curriculum during the study period.

### Intervention

JUMP-in is a school-based intervention primarily aimed at the promotion of sports participation among children in socially and economically deprived areas in Amsterdam. The JUMP-in intervention, targeted sport participation, and outdoor play. The intervention did not directly target screen behaviors. However it was expected that by targeting daily PA, screen behaviors would be influenced as well. JUMP-in was found to be effective in changing organized sport participation [22]. More detailed information concerning the systematic development and design of the intervention can be found elsewhere [21], and is briefly described below.

The Intervention Mapping protocol [23], and RE-AIM framework [24] were applied in order to systematically develop and design the intervention. The intervention was based on the Attitude- Social Influence- self- Efficacy (ASE) model [25], the Environmental Research framework for weight Gain prevention (EnRG) framework [26] and information collected in a pilot study [27,28]. The EnRG framework is a dual process model that combines social cognitive theories (e.g. ASE model [25]) and social-ecological theories (i.e. ANGELO framework [29]). In concordance with the EnRG framework, JUMP-in assumed that behavior is influenced by the environment directly and indirectly, mediated by ASE determinants. The JUMP-in intervention therefore targeted primary schoolchildren’s PA by changing physical, social and political environmental determinants, and cognitive mediators, including social influences, attitude and self-efficacy (see Additional file 1 for an overview of the potential mediators). The JUMP-in is a school-based multicomponent intervention, including 1. Pupil follow up system, a yearly monitoring instruments of PA, BMI and motor skills); 2. School sport activities, daily offer of structural and easily accessible school sport activities in or near the school premises; 3. Calendars offering recurrent breaks for PA, relaxation and posture exercises during regular lessons; 4. Personal workbooks for children and their parents with assignments to perform in class and at home and an instruction book for the school staff; 5. Parental information services including information meetings, courses and sport activities for parents; and 6. Extra care for children at risk, wherein children detected by the pupil follow-up system receive additional adapted physical education lessons or motor remedial teaching. Additional file 1 gives an overview of the hypothesized working mechanisms of the intervention including the potential mediators, intervention strategies, theoretical methods and tools used to change the potential mediators. The intervention duration was 8 months in the first year (from November 2006 to June 2007) and 9 months in the second year (September 2007 to June 2008).

### Measures

Measures were performed at the beginning (T0: September-October 2006) and end of the first school year (T1: May–June 2007) and repeated at the end of the second school year (T2: May–June 2008). Since the implementation of the complete program took more than one school year this study reports on the T0 and T2 measurements. All measurements took place at school and were performed according to standardized procedures by trained testers. *Sports participation* was assessed in a personal interview. Trained interviewers asked whether the child had participated in organized sports activities at least once a week for a minimum of three months (yes or no) directly preceding the interview. Following the results of the pilot study, an interview was the most reliable way to classify sports participation, compared to questionnaires and attendance lists.

*Unorganized outdoor play*, s*creen behaviors* and *mediators* were self-reported in a questionnaire completed in the classroom. Completion took about 45 minutes. The questions concerning outdoor play and screen behaviors were pre-tested and based on previous studies [30-33]. For both variables only the frequency of activities was assessed since children this age are not able to accurately recall the duration of certain activities [34]. Children reported their weekly unorganized *outdoor play* for both summer and winter: How often do you play outdoor?” *never or almost never* [0], *less than once a week* [0.5], *once a week*[1], *14 times per week*[3], *every day or almost every day*[6]. The mean value of winter and summer scores were averaged resulting in a total outdoor play score ranging from 0 to 6.

Leisure time *screen behavior* was determined by assessing the frequency of both weekly TV viewing and computer usage (e.g. gaming, internet, playing “gameboy” etcetera). Since children are better able to recall their activities when a day is divided into parts [32], both TV viewing and computer usage were assessed for three parts of the day: before school, after school and in the evening: How often do you watch TV in the evening? *never or almost never* [0], *less than once a week* [0.5], *once a week*[1], *14 times per week*[3], *every day or almost every day *[6]. Before school, after school and in the evening, scores were summed resulting in a sum score ranging from 0 to 18.

Additional file 1 gives an overview of the hypothesized *personal and environmental mediators* per behavior, including their scales and Chronbach’s alphas. Cronbach’s alphas ranged from 0.65 for cons towards sport participation at baseline to 0.96 for social pressure towards sport participation at 20 months follow-up.

### Statistical analyses

We aim to examine the intervention effect on outdoor play and screen behaviors and to conduct secondary analysis by examining the mediators of the intervention effect on sport participation, outdoor play and screen behaviors. To accomplish our goals, descriptive statistics and t-tests were conducted to examine frequencies of the baseline characteristics and differences between the intervention and control group using SPSS (Version 15.0). The intervention effects on the behaviors were examined with regression analyses using robust maximum likelihood (MLR) estimator in Mplus wherein the behavior was regressed on the intervention condition, controlling for baseline values, covariates and clustering within schools (Muthén and Muthén, Version 6.1). MLR is a maximum likelihood estimator with standard errors and chi-square statistics, that has been shown to be robust to non-normality and non-independence of observations [35].

To test the mediated effects, a multiple mediator path model using MLR estimator was conducted (see Figure 1) informed by the product-of-coefficient test [17], which consists of three steps: 1). Action theory test, which assesses the intervention effect on potential mediator at T2, controlled for the mediator at baseline (T0) (a-coefficient); 2). Conceptual theory, which assesses the association between potential mediators at T2 and outcome variable at T2, controlled for the intervention and baseline values of the mediator and outcome (b-coefficient); and 3). Mediated effect test, wherein the extent of the mediated effect is evaluated, by multiplying the a-coefficient and b-coefficient (a*b coefficient). The path model was developed in two steps. The first step involved testing the factor score of each mediating construct using confirmatory factor analysis, as described below in more detail. The second step involved testing the hypothesized mediators of the intervention on the outcome variable using path modeling. All analyses were adjusted for age, BMI and gender. TV watching and computer use were analyzed in the same model simultaneously. Since mediation can still occur without a significant intervention effect on the outcome [36], mediation analyses were also conducted in absence of a significant main effect.

### Model specification

For all mediating variables measured with more than two items, factor scores were created by conducting confirmatory factor analysis by loading each item on a latent variable and requesting the f-scores. These factor scores are based on the factor loadings of each item and are therefore a kind of weighed sum scores. Factor scores were preferred above mean or sum scores since they control for measurement error. One item measures (e.g. screen behavior mediators) and mean scores of two items measures (e.g. intention) were included as observed variables.

**Figure 2:**
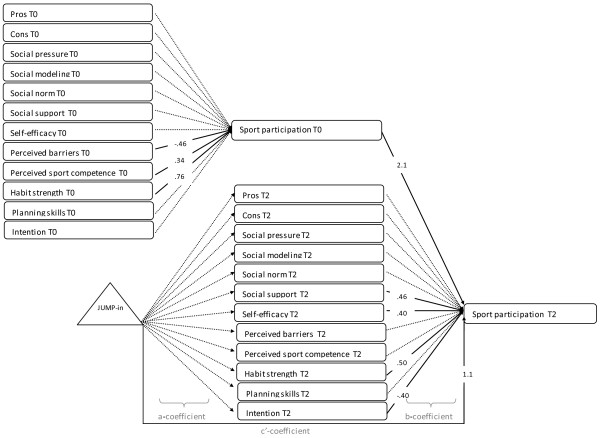
**Path model showing the psychological and environmental mediators of the effect of the JUMP-in intervention on sport participation.** Note: Numbers represent unstandardised regression coefficients. Dotted lines represent non-significant associations, full lines represent significant associations. For reasons of clarity, the model does not show correlations between mediators, the associations between potential mediators at t0 and T2 and the covariates.

**Figure 3:**
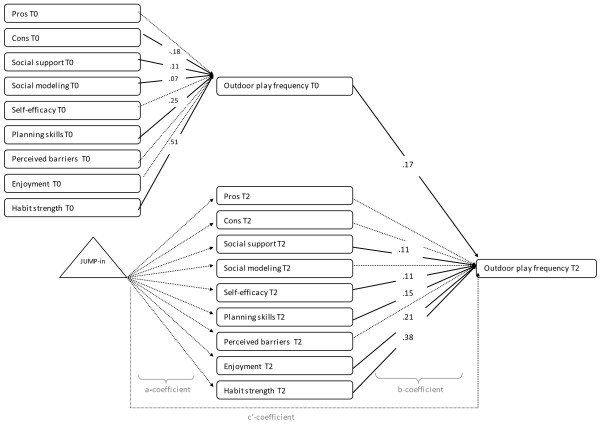
**Path model showing the psychological and environmental mediators of the effect of the JUMP-in intervention on outdoor play.** Note: Numbers represent unstandardised regression coefficients. Dotted lines represent non-significant associations, full lines represent significant associations. For reasons of clarity, the model does not show correlations between mediators, the associations between potential mediators at t0 and T2 and the covariates. Model fit: X2(163)=270.349, p-value=0.00, RMSEA= 0.033 90%CI= 0.026–0.040], CFI=0.933, TLI=0.914.

**Figure 4:**
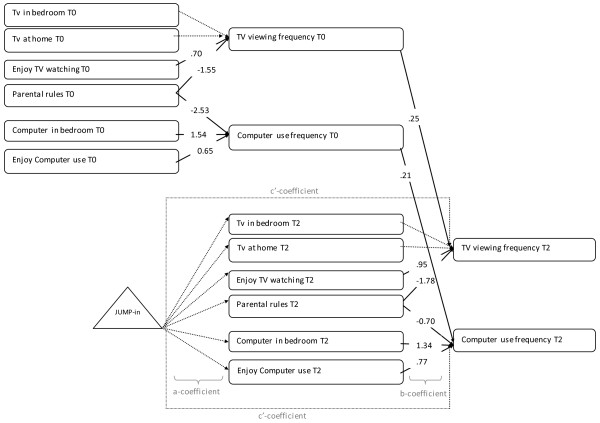
**Path model showing the psychological and environmental mediators of the effect of the JUMP-in intervention on TV viewing and computer use.** Note: Numbers represent unstandardised regression coefficients. Dotted lines represent non-significant associations, full lines represent significant associations. For reasons of clarity, the model does not show correlations between mediators, the associations between potential mediators at t0 and T2 and the covariates. Model fit: X2 (102)= 187.811, RMSEA= 0.037 90% CI= 0.029–0.046], CFI= 0.941, TLI= 0.916.

As seen in Figures 2, 3, and 4, the path models included (1) paths between the potential mediators at baseline (t0) and 20-months (T2) (not shown in figure); (2) paths from the potential mediators to the behavior at baseline; (3) paths from the potential mediators at T2 to the outcome variable at T2 (b-coefficient of mediated effect); and (4) paths between the intervention and measures of the potential mediators at T2 (a-coefficient of mediated effect). The intervention was coded as control (0), or intervention (1) group. There were correlations allowed between the hypothesized mediators at time 0 and time 2 (not shown in figure).

### Model fit

A combination of fit indices was used to determine model fit. A good model fit is indicated by p> .05 for the Chi- square test [37]. Since the Chi-square test is influenced by the sample size, Root Mean Square Error of Approximation (RMSEA), Comparative Fit Index (CFI) and Tucker-Lewis Index (TLI) were calculated to evaluate the model fit. A minimally acceptable fit is obtained when RMSEA < .06, CFI > .95 and TLI > .95 [38]. Chi-square tests were conducted to test for differences between nested models. The fit of the sport participation model could not be calculated using MLR estimator due to the dichotomous outcome but was perceived as acceptable when the goodness of fit indexes using WLSMV estimator (i.e. Weighted Least Square parameter estimator using a diagonal weight matrix with standard errors and mean- and variance adjusted chi-square test statistic [35]) were acceptable using the cut-offs presented above.

## Results

Table 1 shows the baseline values of children's demographics, participation in behavior and mediator values. In total 600 children had complete data on the outcome variables at baseline and T2 (aged 9.8 ± 0.7 years, 51% girls, 13% Dutch ethnicity). Mean BMI was 19.0±3.6, 35% was overweight and 13% obese. At baseline, 41% of the children reported to have participated in sports, and children had played on average 4 times/week (SD= 1.7) outdoors, had watched television (TV) 10 times/week (SD=5.2) and had used the computer 5 times/week (SD=5.1). In the intervention group significantly more children were from a Turkish background, and less children from a Dutch background. In addition, in the intervention group fewer children had participated in sports than in the control group (35 vs. 45%) at baseline.

**Table 1:** Baseline values (mean ± standard deviation or percentages) of demographics, participation in behavior and mediator scores for the total sample, and the control and intervention group separately

**Study characteristics baseline**	**Total**	**Control**	**Intervention**
	**N**=**600**	**N**=**341**	**N**=**259**
*Demographics*			
Age	9.8±0.7	9.8±0.8	9.9±0.7
Gender (%girls)	51%	50%	53%
Ethnicity			
Dutch (%)	13%	16%	9%
Moroccan (%)	37%	36%	39%
Turkish (%)	19%	14%	25%
Surinam/ Antillean (%)	12%	15%	8%
Other, western (%)	6%	8%	5%
Other, non-western (%)	13%	12%	15%
BMI (mean ± SD)	19.0±3.6	18.8±3.6	19.2±3.6
% overweight	35%	34%	36%
% obese	13%	14%	11%
*Behaviors*			
Sports participation (% yes)	41%	45%	35%*
Outdoor play (times/week)	4.1±1.7	4.1±1.7	4.1±1.7
Screen behaviors			
TV viewing (times/week)	10.1±5.2	9.8±5.3	10.3±5.1
Computer use (times/week)	5.4±5.1	5.4±5.1	5.5±5.0
*Mediators- sports*			
Pros [−2,2]	1.2±0.5	0.2±0.5	1.2±0.5
Cons [−2,2]	-.5±0.7	−0.5±0.7	−0.5±0.7
Social modeling [0,4]	2.3±1.1	2.2±1.1	2.3±1.2
Social pressure [−2,2]	0.8±1.1	0.9±1.1	0.8±1.2
Social norm [−2,2]	1.3±0.7	1.3±0.8	1.4±0.7
Social support [0,4]	1.8±1.0	1.8±1.1	1.9±1.0
Self-efficacy [−2,2]	0.0±0.8	−0.0±0.8	0.1±0.8
Sport competence [−2,2]	0.7±0.7	0.7±0.7	0.8±0.7
Perceived Barriers [−2,2]	−0.9±0.8	−0.8±0.8	−0.9±0.8
Intention [−2,2]	0.9±1.0	0.9±1.0	1.0±1.0
Planning skills [−2,2]	0.8±0.8	0.8±0.8	0.9±0.7
Habit strength [−2,2]	0.8±0.9	0.9±0.9	0.8±0.9
*Mediators outdoor play*			
Pros [−2,2]	1.2±0.7	1.2±0.7	1.2±0.7
Cons [−2,2]	-.7±0.9	-.7±0.9	−0.7±0.9
Social support [0,6]	2.5±2.5	2.5±2.5	2.5±2.4
Social modeling [0,6]	2.3±2.3	1.9±2.1	1.8±2.0
Self-efficacy [−2,2]	-.0±0.9	-.1±0.9	0.1±0.9*
Planning skills [−2,2]	0.9±0.8	0.9±0.8	1.0±0.8
Perceived barriers [−2,2]	1.1±0.8	1.1±0.8	1.1±0.8
Enjoyment [0,10]	9.0±1.9	9.0±1.9	9.0±1.9
Habit strength [−2,2]	0.9±0.8	0.9±0.8	0.9±0.9
*Mediators sedentary behavior*			
TV in bedroom (%yes)	52%	50%	54%
# TVs at home	2.3±1.3	2.3±1.3	2.3±1.3
Enjoyment watching TV	7.6±2.5	7.5±2.6	7.7±2.4
Having parental TV rules (%yes)	30%	31%	28%
Computer in bedroom (%yes)	68%	66%	70%
Enjoyment computer use	7.9±2.5	7.8±2.7	8.1±2.3

* p<0.05 intervention group significantly lower than control group.

### Intervention effect on sport participation, outdoor play and screen behavior

Table 2 shows the baseline values, T2 values and adjusted intervention effect on sport participation, outdoor play and screen behaviors. A significant intervention effect on sport participation was found, as intervention children were 2.7 times more likely to participate in sport after the intervention than control children (unstandardized regression coefficient (b)= 0.98, Standard Error (SE)= 0.26; Odds Ratio (OR)=2.68, 95% confidence intervals (95%CI): 1.60, 4.46). No significant intervention effects were found on outdoor play or screen behaviors.

**Table 2:** **Outcome variables at baseline and T2** (**20 months after baseline**) **for control and intervention groups and intervention effect on sport participation**, **outdoor play and screen behaviors**

	**Baseline**		**T2 (20 months)**		**Intervention effect (95% CI)**
	**Control**	**Intervention**	**Control**	**Intervention**	
Sport participation (%)	45%	35%*	48%	62%***	**2**.**68** (**1**.**60**, **4**.**46**)^#‡^
Outdoor play (times/week)	4.1±1.7	4.1±1.7	4.1±1.5	3.9±1.5	−0.30 (−0.79, 0.19)^##^
TV viewing (times/week)	9.8±5.3	10.3±5.1	9.5±4.9	10.2±4.8	0.58 (−0.26, 1.43) ^##^
Computer use (times/week)	5.4±5.1	5.5±5.0	5.5±4.5	6.0±4.7	0.36 (0.35, 1.08) ^##^

* p<0.05 **p<0.01; ***p<0.001 significant difference between intervention and control participants.

^‡^ p<0.001 significant intervention effect.

^#^ Odds Ratio; ^##^ unstandardized regression coefficients.

Analyses were adjusted for school, gender, age, ethnicity, BMI and baseline values.

### Mediated effects

The *sport participation* model depicted in Figure 2 had an acceptable fit (X^2^ (186) = 213.73; p=0.08; RMSEA: 0.016; CFI: 0.982; TLI: 0.960; MLR was used to estimate path coefficients, WLSMV was used to estimate model fit). No significant intervention effect on any of the potential mediators at T2 was found (a-coefficient). Thus no mediators of the intervention effect on sport participation could be identified. However, significant positive associations between social support (b=.46; 95%CI: .04, .88), self-efficacy (b=.41; 95%CI: .15, .66), and habit strength (b=0.50; 95%CI: .14, .86) with sport participation were found (b-coefficients). Changes in intention were negatively associated with sport participation (b=−.40; 95%CI= −.65,-.15).

The *outdoor play* model depicted in Figure 3 represented an acceptable fit (X^2^(163) =270.3, p-value<0.001, RMSEA= 0.033 90%CI= 0.026–0.040], CFI=0.933, TLI=0.914). We found no statistically significant intervention effects on potential mediators at T2 (a-coefficient). Thus no significant mediating effects were identified. However, significant positive associations were found between social support (b=.04; 95%CI: .01-.08), self-efficacy (b=.15; 95%CI: .00-.30), enjoyment (b=.21; 95%CI: .14-.28) and habit strength (b=.38; 95%CI: .18-.58) with outdoor play (b-coefficients).

In the *screen behaviors* model depicted in Figure 4, adding an association between perceived parental TV rules and computer use significantly improved the model (X^2^(2)=11.47; p=0.003). As perceived parental TV rules probably are a proxy for parental rules in general, this association was added. This resulted in a good fit for the screen behavior model (X^2^ (102) = 187.8, RMSEA= 0.037 90% CI= 0.029–0.046, CFI= 0.941, TLI= 0.916). For the screen behaviors model, no effects of the intervention on the potential mediators were found (a-coefficient). Consequently no mediated effects could be identified. However, a significant positive association of enjoying watching TV (b=0.95; 95%CI= 0.75, 1.14) and a negative association of perceived parental TV rules (b=−1.78; 95%CI: -3.01, -.55) with TV viewing were found. In addition, a positive association between enjoying using the computer (b=.77; 95%CI: .56, .94), and having a computer in the bedroom (b=1.34; 95%CI: .62, 2.06) and a negative association from perceived parental TV rules (b=−.70; 95%CI: -1.36, .00) with computer use was identified (b-coefficients).

## Discussion

The JUMP-in study showed a strong intervention effect on sport participation, which confirms previous findings [22]. However, no intervention effect on their hypothesized mediators was found. In addition, no significant intervention effects on outdoor play and screen behaviors or their hypothesized mediators were found.

Despite our finding that none of these mediators were significantly impacted by the intervention, sport participation was positively affected by the intervention. As several hypothesized mediators based on social cognitive models (e.g. pros, cons, intention) were not associated with behavior it suggests that our theoretical assumptions of the intervention were not entirely valid. Thus, other (unmeasured) mechanisms by which the intervention impacted sport participation must be in place. The JUMP-in intervention was based on the EnRG-framework, a dual process model combining social cognitive and social-ecological theories [26]. Based on the EnRG-framework we assumed that by changing the environment we would directly and indirectly (by changing children’s cognitions) change behavior. We therefore targeted several environmental constructs (e.g. organize enjoyable after school sport activities and adapted sport offers) to facilitate participation in organized sport and positively change children’s cognitions towards PA. However, primary schoolchildren’s behavior may be less planned than adults’ behavior and other unconscious/unreasoned processes directly triggered by environmental cues (e.g. availability and parental influences) might influence their behavior [26]. Moreover, primary schoolchildren might have low autonomy, and many decisions regarding their acts are made by their parents. Consequently, the environment might primarily have a direct influence on primary schoolchildren’s behavior instead of an indirect one via cognitive influences. Social cognitive models such as the theory of planned behavior and ASE model as applied and measured in this study, may not fit well for predicting primary schoolchildren’s behavior. As we did not measure the children’s perceived environment, we were not able to assess whether change in environmental constructs yielded by the intervention, directly affected children’s sport participation. Further research on the JUMP-in data, analyzing changes in potential environmental mediators reported by other sources (e.g. parents) should provide more insight into the working mechanisms of the intervention.

Other explanations for the limited intervention effect on any of the potential mediators might be due to unsuccessful intervention strategies. These intervention strategies might simply not have been effective or strong enough to be able to change the potential mediators; they could have mismatched the measured mediating variables, or they were not sufficiently implemented to bring about change in the mediators. Next, as primary schoolchildren have limitations in general cognitive competencies, especially in ability to think abstractly and perform detailed recall, children are less likely to make accurate self-report assessments of past activities and cognitions than adults [30,33]. Other measures such as objective measures or interviews, or combining measures might be more reliable to better characterize primary schoolchildren's activity levels and potential mediators.

The lack of intervention effect on outdoor play, screen behaviors and mediators might also be due to an insufficient implementation of the intervention. Results of the process evaluation [39] showed that JUMP-in is currently embedded in the Amsterdam policy as well as in the organizational structure and daily practices of all participating sectors. However, despite the successful embedding, process data showed some hampering factors of its implementation. An overall impeding factor was the complexity of the multilevel program involving collaborations between multidisciplinary organizations. Consequently, implementers needed more time than expected to synchronize and fine-tune organizational procedures. Further, the comprehensive study measurements took additional time. Two schools decided to postpone the implementation of the in-class lessons. In addition, implementers recommended a simplification of methods, instruments, protocols and tasks of the program components [39]. Lastly, as our a priori power calculation was based on detecting change in sport participation, and not on detecting changes in outdoor play, screen behaviors or any of the potential mediators, our study might have lacked power for detecting change in the other constructs.

The lack of effect on outdoor play and screen behaviors suggests that the school setting might not be the sole channel to influence leisure time activities. As these behaviors are typically performed after school hours, a combination of school-based and family-based intervention strategies may be needed to improve these behaviors, involving the social and physical home environment. JUMP-in did not directly target reducing screen time, but we expected that by targeting outdoor play and sports, screen behaviors would be targeted indirectly. Apparently, this was not the case. This confirms the findings of Biddle and colleagues [40], who examined the temporal patterns of activity and sedentary behaviors in children. They found that TV viewing and sports/exercise participation do not compete for similar time periods on a day but might be able to coexist. This supports the evidence that sedentary behaviors are not just the opposite of PA behaviors and therefore need specific strategies to be influenced.

Still, significant associations between changes in potential mediators (i.e. social support, self-efficacy, habit strength, enjoyment, parental rules, availability and perceived barriers) and changes in behaviors were identified. This confirms the relevance of these constructs in changing these specific behaviors, and that these constructs might be potential mediators. Future intervention studies should search for better or more intensive strategies to affect these potential mediators. The negative association found between intention and sport participation could be explained by the way we measured intention (“Do you intend to *increase* your sport participation within one month?”). We measured intention to change sport participation in stead of intention to participate in sports. Items measuring change are less appropriate measures for mediation analysis. Future studies should take their measures into account when planning to conduct a mediation analysis.

To our knowledge this is the first study examining the mediators of a PA intervention, and the second examining the mediators of screen behaviors in this age group. Importantly, few studies have used appropriate statistical tests to assess mediators in obesity prevention studies [19]. The need for well -conducted mediation analyses in obesity prevention studies has been noted in previous literature [19,41,42]. Our mediation analysis was based on theoretical models such as the EnRG framework and ASE model, providing the opportunity to test these models. In addition the intervention strategies were carefully matched to the targeted mediators, and were tested in a pilot study and adapted based on a process evaluation [21,28]. A final strength was that our program was implemented by the local partners themselves and integrated into a real-world setting, which prevented overestimation of effects due to unrealistic controlled conditions.

Our study was however subject to some potential limitations. First, the measurement of mediators and outcomes relied on child-report. As discussed above, due to limited general cognitive competencies in children, our results may be biased. Future research is need that focuses on the development of (a combination of) valid, reliable and sensitive mediator measures in primary schoolchildren [43]. Second, most of our mediator measures were translated and adapted from existing validated questionnaires because validated Dutch measures were not available, but were not tested for validity or sensitivity. Additionally, to limit participant burden some of the potential mediating variables were assessed by one item, which could have influenced the construct validity and reliability. Next, we assumed a causal association between the potential mediating variables and the outcome variables. We are however aware of the fact that a reciprocal association could exist, wherein changes in the behaviors could have influenced some of the potential mediators. Finally, the process evaluation presented information regarding hampering factors in the implementation and weaknesses in the program strategies. It is impossible to evaluate to what extend these elements were responsible for the lack of change in the mediators.

With these strengths and limitations in mind, future interventions are recommended examining how to effectively improve leisure time behavior such as outdoor play and screen behaviors through school-based interventions. Effective intervention strategies targeting these behaviors should involve the family setting and the physical and social local environment. Other potential strategies include environmental adaptations such as attractive playgrounds, school policy and rules. Actually, these components have been integrated in the recently renewed JUMP-in program. Next, just motivating parents to stimulate and support their children to be physically active, as done in the JUMP-in program, seems not enough. More attention for parental skills is needed in addition to attractive and tailored information. In addition, as suggested by libertarian paternalism, more attention should be paid to the healthy choice as the easy choice in terms of availability, safety and attractiveness of public space to behave physically active [44]. This new perspective has been recently integrated in a new integral healthy lifestyle intervention that focuses on the physical and social environment of primary schoolchildren.

## Conclusions

The JUMP-in intervention was effective in improving sports participation, but not outdoor play, TV-viewing behavior, computer use or any of the potential mediators. Our results show that it is possible to affect leisure time sport participation as part of a school based intervention. However, the lack of mediation findings imply that other (unmeasured) factors operated as mediating mechanisms of the intervention, that we used unsuccessful intervention strategies, that the strategies were inappropriately implemented, or that children are unable to accurately recall past activities and cognitions. Additionally, the school setting might not be the sole channel to influence leisure time activities. Still, several personal, social and physical environmental constructs (social support, self-efficacy, habit strength, enjoyment, parental rules, availability and perceived barriers) were found to be relevant in predicting change in the above mentioned behaviors and seem to be potential mediators. Future interventions are recommended including more effective strategies targeting these relevant constructs, addressing different constructs (e.g. pedagogic skills of parents), and focusing on different implementation settings.

## Abbreviations

ASE-model: Attitude, social influence, self- efficacy model; CFI: Comparative fit index; EnRG framework: Environmental research framework for weight gain prevention framework; MLR: Robust maximum likelihood; PA: Physical activity; RMSEA: Root mean square error of approximation; SB: Sedentary behavior; TLI: Tucker-Lewis index; TV: Television.

## Competing interests

The authors declare that they have no competing interests.

## Authors’ contributions

JDM, MC, MVDW, WVM designed the JUMP-in project. JDM coordinated the implementation of the intervention and the measurements. MVS conducted the statistical analyses and drafted the manuscript. JDM, MC, DK and STV assisted in interpreting the statistical analyses. All authors read and approved the final manuscript.

## Supplementary Material

Additional file 1 Potential mediators, theoretical methods, intervention strategies and materials used in the JUMP-in intervention. Click here for file
