# Supplementary material for: Mediators of the effect of the JUMP-in intervention on physical activity and sedentary behavior in Dutch primary schoolchildren from disadvantaged neighborhoods
Source: Int J Behav Nutr Phys Act. 2012 Nov 6;9:131. doi: 10.1186/1479-5868-9-131 (PMC3541111; doi:10.1186/1479-5868-9-131)
Supplement: Additional file 1 — Potential mediators, theoretical methods, intervention strategies and materials used in the JUMP-in intervention. [file 1479-5868-9-131-S1.doc]

**Additional file 1**: Potential mediators, theoretical methods, intervention strategies and materials used in the JUMP-in intervention.

| **Potential mediator** | **Scale** | **Cronbach’s alpha** | **Theoretical method (theory)** | **Practical strategy/ Intervention strategy** | **Procedures, Material and Provider** |
| --- | --- | --- | --- | --- | --- |
| **Sport participation** | | | | | |
| Pros- Cons | Seven items measuring pros of sports participation (e.g., if I participate in sports I will become stronger), and six items measuring cons of sports participation (e.g., if I participate in sports it will cost me too much time)  Scale: -2= totally disagree to +2= totally agree | Pros:  T0: 0.69  T2: 0.75  Cons:  T0: 0.65  T2: 0.66 | Theory of Planned Behavior  Theory of reasoned action  Persuasive communication | Make children aware of the importance of sports.  Provide children direct positive experiences with sports.  Attitude change through reinforcement,  Repeat positive experiences.  Facilitate sports participation.  Alter physical environments to include more sports options.  Design tailored offers in order to experience desired benefits. | Physical education (PE) teachers talk with children about the importance of sports.  During lessons PE children get acquainted with a variety of attractive sports.  City district and sports associations organize enjoyable after school sports activities and adapted sports offers (moderate intensity, non competitive etc).  Work book-assignment for children to explicate why they do or do not participate in sports. |
| Social modeling | Five items on how much their father, mother, brother(s), sister(s), and friends participated in sports (e.g. My father participates in sports...)  Scale: 0= never to 4= (almost) every day. | T0: 0.67  T2: 0.68 | Social Cognitive Theory | Stimulate child through imitation  Make parents aware of the importance of the parental role model. | Notes for parents in the work book and parental information meetings: ‘Sports and PA behavior is important for your child. Join your child in their leisure time!’ |
| Social norm | Five items on how important their father, mother, brother(s), sister(s) and friends perceived participation in sports (e.g. my father believes that participants in sport is important)  Scale: -2= certainly not to +2= certainly yes | T0: 0.89  T2: 0.90 | Theory of reasoned action  Persuasive communication  Social Cognitive Theory | Make children and parents aware of the importance of sports participation | Information in parental meetings and notes in the les book.  Teachers PE motivate and stimulate inactive children to participate in sports. |
| Social pressure | Five items on whether their father, mother, brother(s), sister(s) and friends believe that (s)he must participate in sports (e.g. My father believes that I have to participate in sports)  Scale: -2= certainly not to +2= certainly yes | T0: 0.94  T2: 0.96 | Persuasive communication  Social Cognitive Theory | Make children and parents aware of reasons to participate in sports / excuses not to sport. | Assignment In the work book to collect all reasons children have regarding not participating in sports and all excuses parents may have to stimulate them (not) to sports. |
| Social support | Six items on the support the child received from family and friends to participate in sports (“how often do your friends ask you to join them in participating in sports?  Scale: 0= Never to 4= (almost) every day. | T0: 0.73  T2: 0.74 | Reinforcement theory  Social Cognitive Theory | Encourage parents to support their children in sports and provide them examples.  Teachers motivate and stimulate children | Notes for parents in “This is the way you Move!” and parental information:  ‘Children need to listen to their body. They all want to learn new movements. Ask them what they want to learn and help them to train these skills.’  ‘Help your child in finding a sport that fits. ‘  Schoolteachers motivate and stimulate children who started sports. |
| Self-efficacy | Eight items measuring the perceived ability to participate in sports in a variety of difficult situations (e.g. do you think it is hard to participate in sports when there is a nice program on TV?)  Scale: -2= very difficult to +2= very easy | T0: 0.79  T2: 0.84 | Theory of Planned Behavior  Reinforcement theory  Task Experience theory  Active learning theory | Tackle barriers and create opportunities for sports participation.  Repeat positive experiences with sports.  Train skills with guided practice and feedback. | Children are offered easy accessible sports offers tailored to their preferences and skills.  Teachers stimulate and support them to participate. |
| Perceived barriers | Nine items measuring whether the child perceived any situational or personal barriers regarding participating in sports (e.g. sport clubs are too far away from my home).  Scale: -2= totally disagree to +2= totally agree. | T0: 0.86  T2: 0.81 | Decision making theory  Theory of reasoned action  Model of physical exercise and habit formation  The Dual Process view | Tackle barriers for sports participation and facilitate participation  Make children aware of hampering factors, remedies and constraints for their personal sports behavior.  Discover what elements/motives attract children to sports and involve these in the strategies.  Inventory perceived hampering factors among children and parents. | Sports are offered:  - Without costs or with very low costs.  - directly after school hours  - in or near the school premises  - tailored to the target group.  Parents are involved in the recruitment of children. |
| Perceived sport competence | Eight items on how much they perceived themselves to resemble the children mentioned in the examples (e.g. some children are very good in a variety of sports).  Scale: -2= I certainly do not, +2= I certainly do too! | T0: 0.77  T2: 0.80 | Reinforcement theory  Active learning. theory | Provide children positive experiences in order to increase perceived competence and self esteem.  Tailor sports offers.  Offer skill training with guided practice and feedback. | Children who are behind in their motor development are offered lessons motor remedial teaching.  PE teachers and trainers in school sport clubs aim to increase perceived sport competence and self esteem. |
| Intention | Two items measuring the intention of changing behavior in the next half year and within the next month respectively.  Scale: -2= certainly no to +2= certainly yes. | T0: 0.73  T2: 0.82 | Model of physical exercise and habit formation,  Theory of Planned Behavior  Theory of reasoned action  Active learning theory  Decision making theory  Social Cognitive Theory | Create easy accessible opportunities for sports participation.  Provide children positive experiences with sports. | Children are offered easy accessible sports offers tailored to their preferences and skills.  Teachers stimulate and support children to participate. |
| Planning skills | Nine items on whether the child finds it difficult or easy to plan sports activities (e.g., do you find it difficult or easy to ask your friend to participate in sports together).  Scale: -2= very difficult to +2= very easy | T0: 0.85  T2: 0.85 | Task Experience theory  Model of physical exercise and habit formation  Theory of reasoned action  Active learning theory | Make children aware of all things they have to organize/prepare for sports participation.  Create opportunities to carry out plans and to evaluate experiences. | An assignment in the work book to write down what sport skills you want to learn, from who, where, what material is needed and how long it will take.  The last chapter is directed to planning skills and self regulation with regard to leisure time sports behavior. Children are asked to fill in a day/week-calendar with all actions they have to plan and realize their own leisure time physical activities. Distinction is made between a school day and a weekend day. |
| Habit strength | Six items on whether sports participation is a habit (e.g. I would find it hard not to participate in sports) Scale: Scale: -2= certainly no to +2= certainly yes | T0:0.82  T2:0.88 | Model of Physical Exercise and Habit Formation  The Dual Process view | Make sports participation part of the weekly routine of the child. | Children are simulated to participate in sports after school time. They are expected to follow the rules regarding presence etc. |
| Preference/ Liking | One item on how much the child likes to participate in a range of activities (e.g. how much do you like the next activities: sports).  Scale: 0 to 10. | - | Active learning theory | Provide children positive experiences with sports. | Children are offered easy accessible sports offers tailored to their preferences and skills. |
| *Outdoor play* | | | | | |
| Pros- Cons | Six items measuring pros of outdoor play (e.g., if I play outdoors I will become stronger), and four items measuring cons of outdoor play (e.g., if I play outdoors do I get injured)  Scale: -2= *totally disagree* to +2= *totally agree* | *Pros*  T0: 0.73  T2: 0.76  *Cons*  T0: 0.71  T2: 0.71 | Theory of Planned Behavior  Theory of reasoned action  Persuasive communication | Make children aware of the importance of daily PA.  Provide children positive experiences with PA. | In the work book attention is directed to the importance of daily PA behavior. Assignments included are;  ‘What does your body need to feel strong/ listen to your muscles; what do you feel when you wake up/after school etc.’  A Quiz contains questions about what a body needs to feel good and be strong.  PE teachers pay extra attention for physical activity games during their lessons PE, which can be played outdoors in the leisure time.  Providing positive experiences with PA is an important priority in lessons PE. |
| Social pressure | One item on how often parents tell the child that (s)he has to play outdoors  Scale: 0= *(almost) never* to 4= *(almost) every day* | - | Social Cognitive Theory  Persuasive communication | Provide parents knowledge regarding importance of daily PA.  Motivate parents to stimulate daily PA behavior of their children. | Notes for parents in “This is the way you Move!” and in the JUMP-in Parental Information Service directed to the importance of the parental role in stimulating children to be daily physically active: “make agreements with your child and remind them to their plans” or “Make sure your child is physically active all days of the week; also on weekend days”.  An assignment for children to note what they want to learn, from whom, where and how long it will take. |
| Social support | One item on how often parents take the child somewhere to play outdoors  Scale: 0= *(almost) never* to 4= *(almost) every day* | - | Social Cognitive Theory    Reinforcement theory | Motivate parents to support daily PA behavior of their children and provide examples. | Information for parents directed to what they can do to support their children in leisure time PA behavior. For example notes in the work book: “Discover the talents and preferences of your child”, “Help your child trying some of the PA ideas he/she has collected at school”, or “Your child is proud of what it can do! Pay attention to that: give a compliment, take a photo and help him/her to get better” or “Your child learns about his /her body. Ask at home what he/she has learned”. |
| Self-efficacy | Eight items measuring the child’s perceived ability to play outdoors in a variety of difficult situations (e.g. do you think it is hard to play outdoors when there is a nice program on TV?)  Scale*:* -2= *very difficult* to +2= *very easy* | T0: 0.82  T2: 0.84 | Theory of Planned Behavior  Reinforcement theory  Task Experience theory  Active learning theory | Tackle barriers and create opportunities for PA.  Provide children positive experiences with PA.  Train PA skills with guided practice and feedback. | Providing positive experiences with PA is a priority in lessons PE. Teachers PE and trainers in school sport clubs aim to decrease feelings of negative perceived sport competence. They encourage children to organize PA games, which they can play in their leisure time.  In “This is the way you Move!” children are encouraged to expose their PA talents; “organize a theatre in which all children present their unique movement-trick (“make a collage from yourselves in your favorite movement”. |
| Panning skills | Six items on whether the child finds it difficult or easy to play outdoors (e.g., do you find it difficult or easy to ask your friend to play outdoors together).  Scale: -2= *very difficult* to +2= *very easy* | T0: 0.79  T2: 0.78 | Task Experience theory  Model of physical exercise and habit formation  Theory of reasoned action  Active learning theory | Make children aware of all things they have to organize/prepare for their daily PA.  Create opportunities to carry out plans and to evaluate experiences. | In the les book the last chapter is directed to planning skills and self regulation with regard to leisure time PA behavior. Children are asked to fill in a day/week-calendar with all actions they have to plan and realize their own leisure time physical activities. Distinction is made between a school day and a weekend day. |
| Perceived barriers | Eight items regarding the availability and possibilities to play outdoors in the child’s neighborhood (e.g. In my neighborhood there is a place where I can play outdoor).  Scale: -2= *certainly not* to +2= *certainly yes.* | T0: 0.82  T2: 0.85 | Model of Physical Exercise and Habit Formation  The Dual Process view  Decision making theory | Make children aware of their social and physical environment (at school, at home and at the route between home and school).  Make children aware of their preferences and wishes regarding environmental factors influencing outdoor play.  Alter physical environments to create more PA options. | In les books attention is directed to awareness of the environment with assignments such as;  Draw the playground at your school/home/friends neighborhood (children can use stickers). Tell about your drawing: what is cool, what should be adapted to make it a better place for outdoor play? |
| Habit strength | Six items on whether outdoor play is a habit (e.g. I find it hard not to play outdoors every day) Scale: Scale: -2= certainly no to +2= certainly yes | T0:0.82  T2:0.89 | Model of Physical Exercise and Habit Formation  The Dual Process view | Make outdoor play part of the weekly routine of the child. | Children are simulated to play outdoors after school time. In one chapter in the les book children fill in their personal favorite PA ideas for playing. |
| Preference/ Liking | One item on how much the child likes to play outdoors (e.g. how much do you like the next activities: outdoor play).  Scale: 0 to 10. |  | Active learning theory | Let children think and tell about their favorite PA activities in their leisure time.  Stimulate them to try new PA experiences. | In one chapter in the les book children fill in their personal favorite PA ideas for playing:   - in all seasons - outdoors - in-class - on your own - together with your parents - to make you feel strong |
| *Screen behavior* |  |  |  |  |  |
| Availability TV in bedroom | One item on whether the child has a TV in bedroom (“Do you have a TV in your bedroom?”)  Scale: 0= no 1= yes | - | Na | Na | None |
| Availability TV at home | One item on how many TVs the child has at home (“How many TVs are available at your home?) | - | Na | Na | None |
| Perceived rules TV viewing | One item on rules at home on time the child is allowed to watch TV (“Are there rules at home about how long you are allowed to watch TV?)  Scale: 0= no 1= yes | - | Na | Na | None |
| Preference TV viewing | One item on how much the child likes to watch TV (e.g. how much do you like the next activities: TV watching).  Scale: 0 to 10. | - | Na | Na | None |
| Availability computer in bedroom | One item on whether the child has a computer in bedroom (“Do you have a computer (or playstation/ x-box) in your bedroom  Scale: 0= no 1= yes | - | Na | Na | None |
| Preference computer use | One item on how much the child likes to use the computer (e.g. how much do you like the next activities: use computer).  Scale: 0 to 10. | - | Na | Na | None |
